# Is the Inclusion of Animal Source Foods in Fortified Blended Foods Justified?

**DOI:** 10.3390/nu6093516

**Published:** 2014-09-04

**Authors:** Kristen E. Noriega, Brian L. Lindshield

**Affiliations:** Department of Human Nutrition, Kansas State University, 208 Justin Hall, Manhattan, KS 66506, USA; E-Mail: klilnor@k-state.edu

**Keywords:** animal source food, fortified blended food, protein, whey protein concentrate, milk, meat, child growth, moderate acute malnutrition, food aid

## Abstract

Fortified blended foods (FBF) are used for the prevention and treatment of moderate acute malnutrition (MAM) in nutritionally vulnerable individuals, particularly children. A recent review of FBF recommended the addition of animal source food (ASF) in the form of whey protein concentrate (WPC), especially to corn-soy blends. The justification for this recommendation includes the potential of ASF to increase length, weight, muscle mass accretion and recovery from wasting, as well as to improve protein quality and provide essential growth factors. Evidence was collected from the following four different types of studies: (1) epidemiological; (2) ASF *versus* no intervention or a low-calorie control; (3) ASF *versus* an isocaloric non-ASF; and (4) ASF *versus* an isocaloric, isonitrogenous non-ASF. Epidemiological studies consistently associated improved growth outcomes with ASF consumption; however, little evidence from isocaloric and isocaloric, isonitrogenous interventions was found to support the inclusion of meat or milk in FBF. Evidence suggests that whey may benefit muscle mass accretion, but not linear growth. Overall, little evidence supports the costly addition of WPC to FBFs. Further, randomized isocaloric, isonitrogenous ASF interventions with nutritionally vulnerable children are needed.

## 1. Introduction

Stunting (length-for-age below 2 SD) and wasting (weight-for-length below 2 SD) affected 165 million and 52 million children, respectively, in 2013 [1]. Moderate acute malnutrition (MAM) is characterized by a weight-for-length *Z*-score (WLZ) >−3 and ≤−2; severe acute malnutrition (SAM) is indicated by a WLZ ≤−3. Micronutrient-fortified blended foods (FBF) are broadly used to prevent and treat MAM in nutritionally vulnerable individuals, particularly children [2]. The most common types of FBF include corn-soy blend (CSB) and wheat-soy blend (WSB) [3].

The United States Agency for International Development (USAID) commissioned a team to analyze current FBF and provide recommendations for improvement [4]. The resulting Food Aid Quality Review (FAQR) recommendations included the addition of 3 g of whey protein concentrate with 80% protein content (WPC80) per 100 g dry FBF [2]. Justification provided in the report for the addition of WPC80 includes: the promotion of linear growth, the accrual of lean tissue, increased protein availability, the provision of essential growth factors, significant nutrient value in a small quantity and a stable price and shelf life [2]. The merit of this justification has been questioned, because: (1) at the time of the report, whey alone had not been studied in nutritionally vulnerable children; (2) interventions that reported increased linear growth used ASF protein doses greater than the proposed rate of 3%; and (3) growth factor evidence was lacking [5]. Prior to these recommendations, a well-conducted review had determined that, at that time, evidence was insufficient to justify the inclusion of whey or skim milk powder in FBF for use by nutritionally vulnerable children [3].

Although FBF are usually blanket-distributed to nutritionally vulnerable regions and given to all household or community members, FBF has the greatest potential to affect the rapid growth of children (birth through adolescence), particularly those who are highly susceptible to or suffering from MAM. The focus of this review is to determine whether sufficient evidence demonstrates that animal source foods (ASF), including milk, whey or meat, improve growth outcomes in nutritionally vulnerable children enough to warrant the inclusion of WPC80 in FBF. Evidence of the additional growth benefits from ASF must clearly offset the increased expense and logistic hurdles of including ASF in FBF.

## 2. Does Evidence Suggest an Additional Growth Benefit from ASF?

### 2.1. Epidemiological Studies

Various epidemiological study designs in the past 40 years have found a positive correlation between ASF consumption and the linear growth and weight gain of nutritionally vulnerable children (Table 1).

**Table 1:** Epidemiological studies of animal source food (ASF) consumption and the growth of nutritionally vulnerable children.

Reference	Location	*n*	Population	Outcome(s)
[6]	Solís Valley, Mexico	67	Enrolled at 18 months, followed 12 months	Weight growth slope correlated to total protein g/day, ASF protein g/day and ASF g/kg. >64 kcal/day of ASF: faster linear and weight growth <64 kcal/day of ASF: faster linear, slower weight growth
[7]	Kingston, Jamaica	191	9–24 months	Average dairy servings/day: stunted—1.5 (0–4.0 range); non-stunted—2.0 (0.5–4.0 range)
[8]	Netherland	243	0–8 year consuming macrobiotic diets	Weight, height and MUAC ↑ with >3 servings/week of dairy *versus* 0–2 servings/week
[9]	Peru	107	Enrolled at 12–15 months, breastfed and weaned, followed 3 months	ASF associated with linear growth when total complementary food intake was low, not high
[10]	Sichuan, China	389	4–12 months	↑ WAZ with liver and blood products >1 serving/week *versus* ≤1 serving/week
[11,12]	Guatemala	263	1. Female adults from a past childhood Atole ^a^ + DSM *vs.* Fresco ^b^ intervention 2. Infants of the female participants	↑ Height of female adults with childhood DSM supplementation *versus* females without childhood DSM supplementation ↑ Birth length in infants born to female adults with childhood DSM supplementation *versus* infants born to females without childhood DSM supplementation

Abbreviations: ASF, animal source food; DSM, dry skim milk; kcal, kilocalories; MUAC, mid-upper-arm circumference; WAZ, weight-for-age *Z*-score. ^a^ A high-energy, high-protein, fortified corn-soy beverage; ^b^ a low-energy sweetened beverage.

Evidence suggests that the growth response from ASF is affected by both the amount of ASF and complementary foods in a child’s diet. In one study, weight gain and linear growth were negatively correlated for toddlers in the lowest quartile of dietary ASF (<61 kcal/day), whereas the outcomes were positively correlated for toddlers with higher ASF intakes (>61 kcal/day) [6]. Lower infant and toddler milk intake (1.5 servings/day), compared with higher intake (2 servings/day), was associated with a significantly higher incidence of stunting [7]. An analysis of the macrobiotic diets of nutritionally vulnerable Dutch children (0–8 years old) found that more than three servings per day of dairy, compared with 0–2 servings per week, was associated with significantly greater height, weight and mid-upper arm circumference (MUAC), an indicator of body mass growth [8]. ASF was associated with significantly increased linear growth in Peruvian toddlers when total complementary food intake was low, but not high [9]. Liver, pork products and total animal protein consumption more than once per week, compared with less frequent consumption, was associated with a significantly higher weight-for-age *Z*-score (WAZ) in nutritionally vulnerable Chinese infants [10]. Milk intake was not a reported outcome in this study, likely because of infrequent consumption in the studied population.

A dual-generational prospective cohort study in Guatemala assessed the long-term effects of ASF in FBF [11]. Female participants of a childhood intervention that compared a dried skim milk beverage to a low-calorie, non-ASF beverage were followed into adulthood [12]. As adults, the women who had received dried skim milk (DSM) not only were significantly taller, but also gave birth to infants with a significantly greater birth length than the non-supplemented group’s infants [11]. After correction for maternal height, however, there was no significant difference in infant length. The positive dual-generational linear growth trend supports consumption of DSM in early childhood and the inclusion of DSM in FBF.

ASF consumption in healthy populations also has been associated with increased growth outcomes. A cross-sectional analysis of healthy Danish children found that height was significantly associated with total protein, animal protein and milk intake, but not with either meat or vegetable protein intake [13]. A study in Iceland found that healthy toddlers with the highest quartile of animal protein consumption, compared with the lowest quartile, had greater weight, height and BMI at 12 months old [14]. A 20-year prospective cohort study in a healthy Danish population found that pregnant women who consumed high amounts of milk (>150 mL/day) produced offspring with significantly greater adult height than mothers who consumed less milk (<150 mL/day) [15]. Maternal milk consumption also has been associated with significantly higher birth weight elsewhere [16], but this relationship is outside the scope of this review.

A limitation of the epidemiological evidence is that it does not allow for the determination of whether the growth outcomes are due to ASF or total dietary protein. It also does not allow for accurate interpretation of whether a specific type of ASF (meat, milk or WPC) is better than other types. Together, the epidemiologic evidence suggests that higher consumption of milk (2–3 servings/day), total animal protein and total dietary protein may contribute to increased linear growth in nutritionally vulnerable and healthy children.

### 2.2. Intervention Trials

A number of intervention trials have reported growth outcomes from ASF interventions in nutritionally vulnerable children. Early trials compared milk with no intervention or a low-calorie control group [12,17,18,19,20,21,22,23,24]. Researchers shifted toward isocaloric interventions [25,26,27,28,29,30], then most recently toward isocaloric, isonitrogenous interventions [31,32,33] that minimize variables and maximize evidence of a direct effect of ASF. These categories will be discussed from weakest to strongest intervention category for the best interpretation of the ASF effect on childhood growth outcomes.

#### 2.2.1. ASF *versus* No Intervention or Low-Calorie Control

ASF interventions, compared with no intervention or a low-calorie control group, have been cited as evidence for the inclusion of ASF, particularly milk, in the diets of nutritionally vulnerable children (Table 2) [13,27,34,35]. DSM, the most commonly studied ASF in non-isocaloric trials, has consistently and significantly increased the length and weight of toddlers [17,18,19,24] and school-aged children [20,21] compared with no other dietary intervention. Evidence suggests that supplementation may have its greatest impact around 12 months of age. Powdered whole milk supplementation more significantly increased linear growth and weight gain at 9–12 months of age compared with 6–9 or 12–36 months of age [19]. In addition, DSM added to a rice-corn-rye-soy cereal resulted in significantly greater linear growth and weight gain in toddlers who enrolled in the study at 12–14 months of age compared with those enrolled at 9–12 months [24].

**Table 2:** Growth effects from animal source food (ASF) *vs.* no intervention or control.

Reference	Location	*n*	Entry Age	Duration	Intervention ^a^	Outcome(s)
[12]	Guatemala	372 homes	6–24 months	3 months	Villages received different beverages, voluntary consumption recorded I. Atole ^b^ + DSM: 163 kcal/drink, 11.5 g II. Fresco ^c^: 59 kcal/drink, 0 g	Wasting: high participation/consumption with Atole (≥10% energy Reference Daily Intake) increased recovery rate
[17]	Guatemala	453	Birth	3 years	Villages received different beverages, voluntary consumption recorded I. Atole ^b^ + DSM: 163 kcal/drink, 11.5 g II. Fresco ^c^: 59 kcal/drink, 0 g	Height, weight: Atole > Fresco; LBM: Atole increased calf circumference and female MUAC and MAMA
[18]	Colombia	131	Birth	3 years	Daily family intervention ^d^ I. DSM, enriched bread, vegetable oil: 3–5 months, 670 kcal/day, 30.2 g; 6–11 months, 428 kcal/day, 22.7g; 12–36 months, 623 kcal/day, 30 g II. Control, no intervention	Height, weight: milk > control growth rates
[19]	Colombia	232	Birth	3 years	Daily I. Whole powdered milk and vegetable mix: 3–6 months, 670 kcal/day, 22.7 g; 6–12 months, 428 kcal/day, 22.7 g; 12–36 months, 623 kcal/day, 30 g II. Control, no intervention	Height, weight: milk > control; absolute responsiveness greatest at 3–6 months; growth responsiveness greatest at 9–12 months
[20]	Bundi, New Guinea	86	7.7–13 years	8 months	5 days/week, skim milk powder with water or meal I. Control, no intervention II. 10: 98 kcal/day, 10 g III. 20: 201 kcal/day, 20 g	Weight: 20 g > 10 g; height: no difference between interventions TSF and SSF: control > both interventions
[21]	Vietnam	444	7–8 years	6 months	6 days/week, 250 mL servings/twice each day I. UHT-whole milk: 77 kcal/100 g, 3 g/100 g II. Fortified UHT-whole milk: 75 kcal/100 g, 3.2 g/100 g III. Control, no intervention	Height, weight, % underweight, % stunted: milk groups > control
[22]	Guatemala	n/a	6–48 months	3 or 6 months	Villages received different beverages, voluntary consumption recorded. Non-wasted children I. Atole ^b^ + DSM: 163 kcal/drink, 11.5 g II. Fresco ^c^: 59 kcal/drink, 0 g	Wasting: 3 and 6 months of Atole prevented the onset of wasting; effects greater in children with lower initial WLZ
[23]	Netherland	209	7–17 years (10.9 mean)	6 years	Parents of stunted children with macrobiotic diets given dietary recommendations, including increase dairy consumption. No control	Meat and dairy added to diets. Girls: height, weight, MUAC; dairy > (dairy + egg + meat + fish) Boys: no direct relationship with ASF
[24]	Ecuador	110	9–14 months	11 months	5 day/week, non-randomized I. Mi Papilla ^e^ + DSM: 275 kcal/day, 10 g II. Control, no intervention	Height, weight, % underweight; Mi Papilla > control; effects greater in children with an older enrollment age (12–14 months)

Abbreviations: DSM, dried skim milk; kcal, kilocalories; SSF, subscapular skinfold; TSF, triceps skinfold; UHT, ultra-heat-treated; WLZ, weight-for-length *Z*-score; MAMA, mid-upper arm muscle area. ^a^ Information represents the distributed amount of each intervention and does not reflect actual consumption. Calories (kcal/day) and protein (g) are indicated after each intervention; ^b^ a high-energy, high-protein, fortified corn-soy beverage; ^c^ a low-energy sweetened beverage; ^d^ in addition to the child supplementation, the mothers received intervention food during the third semester prior to the participants’ births; ^e^ a rice-corn-rye-soy cereal; group selected from poorer communities; control selected from wealthier communities.

DSM, when compared to no intervention or a low-calorie control, may increase lean body mass (LBM), which can be measured by skinfold measurements, an indication of subcutaneous tissue and body fat, or mid-upper arm measurements, an indication of muscle composition [36]. When compared to no intervention, 10 g and 20 g of DSM did not increase triceps or subscapular skinfolds of school-age children [20]. In another study, Atole, a corn-soy beverage with DSM, compared to Fresco*,* a low-calorie beverage, significantly increased male and female toddler calf circumference, female MUAC and female mid-upper arm muscle area (MAMA) [17].

A lower incidence of wasting, stunting and being underweight has been associated with ASF. Supplementation with the same Atole (DSM) beverage, compared with the lower-calorie Fresco, significantly prevented [22] and increased recovery from [12] MAM wasting in Guatemalan toddlers, but the size of the studied population was not described [22]. Stunting and underweight incidence in Vietnamese children was significantly reduced with whole milk supplementation compared with no intervention [21].

Children who are nutritionally vulnerable because of diet choices also have been studied. Researchers examined the growth effects of incorporating milk, meat, fish and eggs into macrobiotic diets [23]. Parents were given dietary recommendations to add ASF, particularly milk, into the diets of their stunted children (0–8 years old). Six years after these recommendations, participants had increased their consumption of meat and milk. There was no significant relationship between ASF consumption and male growth, but milk alone or in combination with other ASF was associated with significant improvements in female length-for-age *Z*-scores (LAZ), WLZ and MUAC. Meat, fish or egg, either alone or in combination, were not associated with increased growth for males or females [23].

Compared with no intervention or a low-calorie control group, ASF has consistently increased length, weight and LBM; it has also decreased the incidence of MAM wasting, stunting and being underweight. These outcomes, however, could be attributed to a number of variables, including, but not limited to: ASF, DSM or increased total caloric or protein intake. Higher energy consumption clearly corresponds to an increase in all growth outcomes in nutritionally vulnerable children.

#### 2.2.2. ASF *versus* Isocaloric Non-ASF

Six trials compared ASF to an isocaloric non-ASF intervention (Table 3) [25,26,27,28,29,30]. Every intervention, unless otherwise specified below, was FBF. All trials that reported weight outcomes found no significant weight gain benefit from the ASF intervention [25,26,27,28,29]. Because of different growth rates throughout childhood, linear and LBM outcomes will be addressed by the enrollment age of study participants (toddlers and school-aged children).

**Table 3:** Growth effects from isocaloric animal source food (ASF) *vs.* non-ASF interventions.

Reference	Location	*n*	Entry Age	Duration	Intervention ^a^	Outcome(s)
[25]	Bundi, New Guinea	88	5.5–15.5 years	13 weeks	5 day/week (270 kcal/day) I. Control, no intervention II. Skim milk powder drink: 27.12 g * III. Extra margarine in meal IV. 5 meals/day instead of the normal 3 (actual consumption not monitored)	Weight: milk and margarine, no difference between interventions; height: milk > margarine; SSF: milk > margarine
[26]	Ghana	190	6 months	6 months	500 g/week distributed to mothers to feed ≥3×/day (310 kcal/day) ^b^ I. Weanimix ^c^: 10.7 g II. Weanimix + vitamins + minerals: 0.7 g III. Weanimix + fish powder: 20 g IV. Koko ^d^ + fish powder: 17.9 g V. No Intervention	Weight, height, MAMA: no difference between interventions
[27,37,38]	Embu District, Kenya	910	6–14 years	2 years	5 day/week during school year (Cohort I, 240 kcal/day; Cohort II, 313 kcal/day) I. Githeri ^e^ + minced beef: 19.2 g; 21.7 g II. Githeri + UHT whole milk: 12.7 g; 15.2 g III. Githeri + oil: 7.9 g; 8.4 g IV. Control	Weight, height: no difference between interventions; MAMA: meat > energy; no difference between milk and energy; MUAC: no difference between interventions
[28]	Embu District, Kenya	554	11–40 months	5 months	5 day/week (270 kcal/day) I. Porridge ^f^ + UHT whole milk: 5.9 g II. Porridge + minced beef: 13.0 g III. Porridge + added oil and sugar: 3.4 g	Weight: no difference between interventions; height, MUAC: milk > meat; no difference between milk and energy or meat and energy; MAMA: energy > meat; no difference between energy and milk
[29]	Republic of Congo; Zambia; Guatemala; Pakistan	1062	6 months	12 months	Daily (70 kcal/day 6–11 months; 105 kcal/day 12–18 months) I. Lyophilized beef: 13 g; 19.5 g II. Cereal ^g^: 3 g; 4.6 g	Weight, height, stunting rate, wasting rate: no difference between interventions
[30]	China	1465	6 months	12 months	Daily (148 kcal/day) I. Pork: 12.8 g * II. FC ^h^ cereal III. Rice ^i^	Height: meat > cereal

Abbreviations: DSM, dried skim milk; kcal, kilocalories; MAMA, mid-upper-arm muscle area; MUAC, mid-upper-arm circumference; SSF, subscapular skinfold; UHT, ultra-heat-treated. ^a^ Information represents the distributed amount of each intervention and does not reflect actual consumption. Protein (g) is indicated after each intervention; ^b^ nutrient information was reported per kg of intervention food. Based on amount distributed per week (500 g), daily values were calculated; ^c^ a corn-soy-peanut cereal mix; ^d^ a low-energy, low-nutrient fermented traditional weaning food, 276 kcal/day; ^e^ a local maize-bean dish; ^f^ millet-based porridge; ^g^ rice-soy cereal; ^h^ fortified-cereal-based supplement; ^i^ non-fortified rice supplement; * calculated from the United States Department of Agriculture National Nutrient Database: 75 g of skim milk powder was calculated to have 27.12 g of protein.

Of the four toddler trials (six months to three years), three reported no difference in growth outcomes between the ASF and non-ASF interventions [26,29]. A well-controlled trial found no significant difference between lyophilized beef (non-FBF) and rice-soy cereal interventions for linear growth or recovery from stunting and wasting [29]. The second study reported no linear growth or LBM difference from interventions with or without fish powder, with different base cereals (a corn-soy-peanut cereal and a traditional low-calorie fermented weaning food) or with or without vitamin and mineral fortification [26]. Although the interventions were isocaloric, the multiple variables limit the interpretation of the results. The third study, a millet-based porridge intervention, compared the addition of oil, minced beef and ultra-heat-treated whole milk (UHT-milk). Oil, compared to meat, significantly increased MAMA; UHT-milk, compared to meat, significantly increased MUAC [28]. There were no significant LBM differences between the UHT-milk and oil. LAZ and linear growth significantly improved with UHT-milk supplementation compared with minced beef. There was no linear growth difference between UHT-milk and oil or minced beef and oil. It should be noted that meat participants consumed significantly fewer daily and intervention calories than the oil intervention group.

The fourth toddler trial reported growth differences between ASF and non-ASF supplementation [28,30]. This trial found that pork (non-FBF) significantly increased the linear growth rate compared with two non-ASF interventions, a fortified cereal (details not available) and a non-fortified rice [30]. To date, however, only an abstract has been released.

Two trials enrolled school-aged children (5.5–15.5 years) [25,27]. In the first, skim milk powder, compared with margarine or additional taro-sweet potato meals, significantly increased linear growth and subscapular skinfolds [25]. In the second study, UHT-milk, minced beef or extra oil was added to a maize-bean dish. No significant differences were detected between interventions for linear growth or MUAC outcomes, but minced beef significantly increased MAMA compared with oil [27,37,38]. It should be noted that a persistent and severe regional drought caused food shortages for the duration of this study that may have inhibited the overall growth of the children.

Evidence from isocaloric interventions suggests that skim milk powder or UHT-milk supplementation, compared with oil or energy, may be beneficial for LBM, but evidence that milk increases linear growth is limited. No milk protein dose-response relationship has been identified. Evidence that meat supplementation increases growth outcomes in nutritionally vulnerable children compared with isocaloric non-ASF or milk interventions is limited.

#### 2.2.3. ASF *versus* Isocaloric, Isonitrogenous ASF

None of the identified isocaloric, isonitrogenous trials intervened with traditional FBF, so a brief explanation of the different interventions follows. FBF replace or supplement local dishes to support health and growth, whereas ready-to-use supplementary foods (RUSF) and fortified spreads are complementary foods designed to treat MAM [39]; they are used for a shorter duration, are more energy- and nutrient-dense and are typically more expensive. Ready-to-use therapeutic foods (RUTF), the most energy- and nutrient-dense formulation, therapeutically treat SAM and are intended as the main, if not only, source of energy during a shorter treatment period [39].

Two trials that compared isonitrogenous ASF and non-ASF interventions included a third intervention with a corn-soy blend (CSB) (Table 4) [32,33]. The first trial compared a soy-peanut-fortified spread (18.9 g protein), a DSM-peanut-fortified spread (18.9 g protein) and a non-isonitrogenous, non-ASF CSB (34.4 g protein) [32]. The fortified spreads equally and significantly increased weight and MUAC better than CSB and had significantly better MAM recovery rates. No significant additional benefit from the DSM was detected.

The second trial compared soy-whey RUSF (15 g protein), a near-isonitrogenous soy-RUSF (17 g protein) and CSB++ (21 g protein) [33]. CSB++ is a newer corn-soy blend that contains DSM. The CSB++ contained four times less animal protein than soy-whey RUSF, and although not a limiting factor, its lower energy density and added water during preparation required the consumption of more than eight-times the quantity of the RUSF to provide an equal amount of protein and energy. The only benefit to whey supplementation was a significantly greater MUAC increase compared with soy RUSF and CSB++, which did not differ from each other. No significant difference was detected between any intervention for the linear growth rate or wasting recovery rate (the percentage of the group recovered). Both RUSF groups gained significantly more weight than CSB++ participants, recovered from MAM wasting significantly earlier and developed significantly less SAM.

A third isonitrogenous trial examined the efficacy of two soy-based RUTF (15 g protein) with different concentrations of DSM, 10% and 25%, for the treatment of SAM [31]. The 10% DSM intervention, compared with the 25%, had a significantly lower SAM recovery rate and was significantly less effective for weight gain, linear growth and MUAC gain. Omission of a non-ASF intervention limits the interpretation of these results.

Isocaloric, isonitrogenous interventions have provided further insight into the effects of ASF on growth outcomes. DSM or whey provided no additional weight or linear growth benefit compared with an isonitrogenous, non-ASF intervention. Whey supplementation, compared with isonitrogenous soy supplementation, may increase MUAC; however, because this is the only trial to study whey supplementation in nutritionally vulnerable populations, additional evidence is necessary to determine its efficacy in FBF.

**Table 4:** Growth effects from isocaloric, isonitrogenous animal source food (ASF) *vs.* non-ASF interventions.

Reference	Location	*n*	Entry Age	Duration	Intervention ^a^	Outcome(s)
[31]	Malawi	1874	6–59 months	≤8 weeks	Severely wasted children (175 kcal/kg·day) I. 25% DSM RUTF ^b^: 5.49 II. 10% DSM RUTF: 5.49	Height, weight, MUAC: 25% > 10% Wasting recovery rate: 25% > 10%
[32]	Malawi	1302	6–60 months	≤8 weeks	Moderately wasted children (75 kcal/kg·day) I. DSM + FS ^c^: 1.89 II. Soy + FS: 1.89 III. CSB: 3.44	Height: no difference between interventions; weight, MUAC: both FS > CSB; wasting recovery rate: both FS > CSB; CSB recovery occurred later
[33]	Malawi	2712	6–59 months	≤12 weeks	Moderately wasted children (75 kcal/kg·day) I. CSB++ ^d^: 2.8 II. Soy RUSF: 2.26 III. Soy-whey RUSF: 2	Height: no difference between interventions; weight: both RUSFs > CSB++ MUAC: soy-whey > soy and CSB++; wasting recovery rate: no difference between intervention; CSB++ recovery occurred 2 days later; significantly more CSB++ developed SAM

Abbreviations: CSB, corn-soy blend; DSM, dried skim milk; FS, fortified spread; kcal, kilocalories; MUAC, mid-upper-arm circumference; RUSF, ready-to-use supplementary food; RUTF, ready-to-use therapeutic food; SAM, severe acute malnutrition. ^a^ Information represents the distributed amount of each intervention and does not reflect actual consumption. Protein (g/kg·day) is indicated after each intervention; ^b^ soy-based; ^c^ peanut-based fortified spread; ^d^ a corn-soy blend with dried skim milk.

## 3. Additional Justification for the Inclusion of ASF in FBF

The FAQR included two additional justifications for the inclusion of ASF in FBF: improved protein quality and provision of essential growth factors [2].

### 3.1. PDCAAS Value

According to the 2011 FAQR, the Protein Digestibility-Corrected Amino Acid Score (PDCAAS) of CSB will increase from 0.85 to 0.88 with the addition of 3% WPC80 by weight [2]. PDCAAS, the currently accepted measurement of protein quality based upon amino acid content and digestibility, is indicative of the amount of protein and its bioavailability. Foods with a PDCAAS greater than 0.80 are considered good protein sources [2]. The Food and Agriculture Organization of the United Nations (FAO) identified three major limitations of the PDCAAS method: (1) overestimation of amino acid absorption; (2) truncation of the score at 1.00; and (3) overestimation of bioavailability [40]. As indicated in a comprehensive PDCAAS review, legume and cereal antinutritional factors—trypsin inhibitors, tannins and phytates—may reduce amino acid digestibility by up to 50% and protein quality by up to 100% [41]. Moreover, the FAO is moving toward a new method of protein quality determination, the Digestible Indispensable Amino Acid Score (DIAAS), which is expected to better account for the PDCAAS method limitations [40,41]. No evidence was found or included in the FAQR to indicate that the recommended 3% increase in PDCAAS is meaningful enough to increase growth outcomes. Therefore, the recommendation to include WPC80 in FBF, as justified by a 3% increase in PDCAAS, currently lacks evidence.

### 3.2. Growth Factors

It is postulated that childhood growth is increased by certain milk components, including growth factors, lactoferrin, bioactive factors, milk peptides and lactose [35,42,43]. The term “growth factors” loosely refers to the grouping of hormones, cytokines and specific proteins, such as insulin-growth factor-1 (IGF-1), that are involved in cellular growth and repair [44]. The FAQR identified IGF-1 as the “essential” growth factor of interest to increase the potential of FBF to effectively manage wasting and promote linear growth [2].

Milk is generally accepted to stimulate circulating IGF-1, which may, in turn, increase linear growth [3,34,44]. Regular consumption of milk or animal protein, but not meat, has been positively associated with increased serum IGF-1 [13]. A recent study also associated dairy protein intake with serum IGF-1 levels in six-year-old girls [14]; however, seven-day supplementation of casein, but not whey, increased serum IGF-1 in healthy Danish boys [45]. Without the synergistic effect of all milk components, whey’s individual effect on IGF-1 and subsequent growth remains unsupported [3,43].

Exercise science researchers have further studied the differential effects between whey and casein, with a focus on their amino acid profiles. Overall, whey improves muscle performance and is absorbed more rapidly than casein, but no difference in muscle uptake or satiation has been found [46].

## 4. Conclusions

The merit of the recommendation to include WPC80 in FBF was questioned based upon three criticisms [5]:

(1) At the time of the report, whey alone had not been studied in nutritionally vulnerable children. Matilsky *et al.* [32] (2009) had, in fact, studied whey in nutritionally vulnerable children prior to publication of the FAQR. This trial was mentioned in the FAQR, but its findings were not used as supportive evidence for the report’s recommendations, likely because the intervention food was a fortified spread instead of an FBF. No other trials with whey and nutritionally vulnerable children have been identified.
(2) Interventions that reported increased linear growth used ASF protein doses greater than the proposed rate of 3%. To include WPC80 at 3% by weight of CSB would provide 2.4 g of animal protein, accounting for 13% of the total recommended 18 g of protein [4]. The isocaloric trials included in this review that provided sufficient data for calculation used ASF protein doses from 46.5% to 100% [26,27,28,29,30]. The two isocaloric, isonitrogenous studies for which a protein dose could be calculated ranged from 13% to 60% [32,33]. The whey RUSF that increased MUAC had an ASF protein dose of 13% [33]; thus, further investigation is warranted to determine whether the recommended amount of WPC80 would improve growth outcomes.
(3) Growth factor evidence was inadequate at the time of publication. Although research exists on growth factors, specific evidence of whey’s effect on the growth of nutritionally vulnerable children by means of growth factors remains unsupported [3,35,43]. In addition, it should be mentioned that higher protein intake in infancy may increase later obesity risk [47], an effect that may be mediated through insulin and IGF-1 [48].

The focus of this paper was to review whether there is sufficient evidence that ASF increases growth outcomes in nutritionally vulnerable children. Evidence from all of the intervention trials is summarized in Table 5. Epidemiological studies consistently associated improved growth outcomes with ASF consumption; however, there is little evidence from isocaloric and isocaloric, isonitrogenous intervention studies to support the inclusion of meat or milk in FBF. Whey may benefit muscle mass accretion, but not linear growth. The move toward isocaloric, isonitrogenous studies will provide further insight into the extent of milk’s impact on growth. The FAQR authors’ response to criticism is most relevant for considering the addition of ASF to FBF: “The critical metric is not cost per ton of product … but rather cost per impact or effect” [49]. Overall, we conclude from the ineffectiveness of ASF and whey in isocaloric and isocaloric, isonitrogenous intervention studies that the addition of whey and ASF would not positively influence the cost per impact or the effect of FBF.

**Table 5:** Summary of interventions: did the animal source food (ASF) have a better growth outcome than the non-ASF?

Study	ASF	Height	Weight	MUAC	MAMA	TSF	SSF	Wasting Rr
ASF *vs.* Control/No Intervention								
[12]	DSM							+
[17]	DSM	+	+	+f	+f			
[18]	DSM	+	+					
[19]	Whole powdered milk	+	+					
[20]	Skim milk powder	−	+			+	+	
[21]	UHT-whole milk	+	+					
[22]	DSM							+(prevention)
[23]	Meat and dairy	+f	+f	+f				
[24]	DSM	+	+					
ASF *vs.* Isocaloric Non-ASF								
[25]	Skim milk powder	+	−				+	
[26]	Fish powder	−	−		−			
[27,37,38] **	Minced beef	−	−	−	+			
[27,37,38] **	UHT-whole milk	−	−	−				
[28] **	Minced beef	−	−		*			
[28] **	UHT-whole milk	−	−	−	−			
[29]	Lyophilized beef	−	−					−
[30]	Pork	+						
ASF *vs.* Isocaloric, Isonitrogenous Non-ASF								
[32]	DSM	−	−	−				−
[33]	Whey	−	−	+				−

Abbreviations: ASF, animal source food; DSM, dried skim milk; MAMA, mid-upper arm muscle area; MUAC, mid-upper arm circumference area; Rr, recovery rate; SSF, subscapular skinfold; TSF, triceps skinfold; UHT, ultra-heat-treated; +, the ASF had a better growth outcome than the non-ASF; −, no difference between the ASF and non-ASF; +f, the ASF had a better growth outcome than the non-ASF for females; * the non-ASF had a better outcome than the ASF; ** each ASF intervention is included in the table separately; thus, this study is included twice.
